# Comparison of adjuvant and neoadjuvant chemotherapy in the management of advanced ovarian cancer: a retrospective study of 574 patients

**DOI:** 10.1186/1471-2407-6-153

**Published:** 2006-06-08

**Authors:** Arturas Inciura, Andrius Simavicius, Elona Juozaityte, Juozas Kurtinaitis, Ruta Nadisauskiene, Eimantas Svedas, Skirmantas Kajenas

**Affiliations:** 1 Kaunas university of Medicine, Eiveniu 2, LT-50009 Kaunas, Lithuania; 2 Clinic of Obstetrics and Gynaecology, Šiauliai hospital, Architektu 75, LT-78170 Šiauliai, Lithuania; 3 Oncology Institute of Vilnius University, Polocko 2, Vilnius, Lithuania

## Abstract

**Background:**

There is a lack of clinical data on the validity of neoadjuvant chemotherapy in the treatment of ovarian cancer. The aim of this study was to compare the impact of the adjuvant and neoadjuvant chemotherapy regimens on the clinical outcomes in patients with advanced ovarian cancer.

**Methods:**

We performed a retrospective analysis of 574 patients with advanced ovarian cancer admitted to four Lithuanian oncogynaecology departments during 1993–2000. The conventional combined treatment of cytoreductive surgery and platinum-based chemotherapy was applied to both the group that underwent neoadjuvant chemotherapy (n = 213) and to the control group (n = 361). The selection criterion for neoadjuvant chemotherapy was large extent of the disease. Overall and progression-free survival rates and survival medians were calculated using life tables and the Kaplan-Meier method.

**Results:**

There was no difference in median overall survival between stage III patients treated with adjuvant chemotherapy and neoadjuvant chemotherapy (25.9 months *vs*. 29.3 months, *p *= 0.2508) and stage IV patients (15.4 months *vs*. 14.9 months, *p *= 0.6108). Similarly, there was no difference in median progression-free survival between stage III patients treated with adjuvant chemotherapy and neoadjuvant chemotherapy (15.7 months *vs*. 17.5 months, *p *= 0.1299) and stage IV patients (8.7 months *vs*. 8.2 months, *p *= 0.1817). There was no difference in the rate of the optimal cytoreductive surgery between patients who underwent the neoadjuvant chemotherapy and patients primarily treated with surgery (n = 134, 63% *vs*. n = 242, 67%, respectively).

**Conclusion:**

There was no difference in progression-free or overall survival and in the rate of optimal cytoreductive surgery between the neoadjuvant and adjuvant chemotherapy groups despite the fact that patients receiving neoadjuvant chemotherapy had a more extensive disease. Multivariate analysis failed to prove that neoadjuvant chemotherapy could be considered as an independent prognostic factor for survival, and the findings need to be investigated in the future prospective randomised studies.

## Background

Ovarian cancer is the leading cause of death among all gynaecologic cancers in Europe and the United States. The incidence of ovarian cancer has been steadily increasing over the past 10 years in many countries, reaching the overall lifetime risk of 1.8% [1]. Despite new medical and surgical advances and new chemotherapeutic regimens, the overall 5-year survival for patients with stage III and IV epithelial ovarian cancer has remained relatively unchanged over the last 40 years. After the application of cytoreductive surgery and adjuvant chemotherapy with cisplatin/cyclophosphamide, the 5-year survival among stage III cases is 10–20%, and among stage IV cases – even below 10%. The optimisation of the treatment is the only way to prolong the survival of such patients.

Frei in 1982 originally introduced the definition of neoadjuvant chemotherapy to describe chemotherapy treatment of primary solid tumours before surgical ablation. Tumour downstaging has been demonstrated for a variety of tumour types after neoadjuvant chemotherapy. Unlike most solid tumour types, resection of metastatic sites from ovarian cancer is an accepted practice. It was assumed that optimal cytoreductive surgery could be performed in only about 50% of patients with ovarian cancer stages III or IV. In this group of patients, neoadjuvant chemotherapy was applied as an alternative to conventional surgery. Neoadjuvant chemotherapy resulted in adequate tumour shrinkage and allowed for the surgical treatment of tumours previously considered to be unresectable. Neoadjuvant chemotherapy could lead to optimal conditions for cytoreductive surgery and further increase the survival rate. There is a number of assumed advantages and potential disadvantages of neoadjuvant chemotherapy. The proposed benefits include tumour downstaging, which allows for more conservative surgery or more optimal cytoreductive surgery for unresectable tumours. The potential disadvantages include the selection of resistant tumour cell clones, less complete tumour downstaging, and higher local relapse as a result of more conservative surgery and compromised wound healing. Retrospective data support a survival benefit to maximum tumour reduction in advanced stage ovarian cancer. Bristow RE et al. in a meta-analysis of 6885 patients demonstrated a significant positive correlation between the percentage of maximal cytoreduction and a log median survival time [2]. However, 50 to 60% of patients with advanced disease are not optimally debulked [3]. For this subset of patients, the overall prognosis is worse despite significant heterogeneity in tumour chemosensitivity.

Optimal surgery after neoadjuvant chemotherapy is feasible for 60–94% of patients with advanced ovarian cancer [4-8]. The responses of ovarian cancer to neoadjuvant chemotherapy vary from 60% to 80%. This allows for predicting chemosensitive tumours after neoadjuvant chemotherapy. Patients with tumours sensitive to chemotherapy can be treated with optimal cytoreductive surgery, and increased overall and progression-free survival can be expected. In contrast, the feasibility of optimal surgery and the prognosis of patients with chemoresistant tumours are lesser.

The results of studies on neoadjuvant chemotherapy are presented in Table 1. In many studies, surgery was performed after 3 to 4 chemotherapy cycles due to smaller chemotherapy-induced fibrosis compared to fibrosis that develops after six courses of chemotherapy. Earlier studies showed that second-look surgery after 6 cycles of chemotherapy did not increase the survival of patients.

Recently, the efficiency of neoadjuvant chemotherapy in ovarian cancer treatment has been widely discussed, but data for the legitimation of this treatment technique are not sufficient. We found that it is important to analyse and share experience in clinical practice due to the favourable situation in Lithuania where there is already a formed tradition to apply neoadjuvant chemotherapy.

The aim of this study was to evaluate the role of neoadjuvant chemotherapy in the management of advanced ovarian cancer compared to adjuvant chemotherapy, and to determine the impact of the neoadjuvant chemotherapy on the optimisation of cytoreductive surgery.

## Methods

The study included 574 patients with advanced ovarian cancer (FIGO stages III – IV) treated in four Lithuanian oncogynaecology departments during 1993–2000. The information about the patients for this study was obtained from medical records. All the studied patients underwent combined treatment of cytoreductive surgery and chemotherapy CP scheme (*Cisplatin *75 mg/m^2 ^+ *Cyclophosphamide *750 mg/m^2 ^every 3 weeks). The patients were treated with neoadjuvant chemotherapy (3 cycles of chemotherapy, cytoreductive surgery, and 3–5 cycles of chemotherapy) or adjuvant chemotherapy (cytoreductive surgery and 6–8 cycles of chemotherapy). Clinical findings and diagnostic imaging findings were used to decide whether to start the treatment with surgery or with chemotherapy. The selection criterion for neoadjuvant chemotherapy was the large extent of the disease – large volume of ascites, and large tumours in pelvis or abdomen detected by palpation or radiologically. The optimal cytoreduction in this analysis was considered to be achieved when the largest tumour diameter of residual disease was less than 2 cm. Patients treated with 6–8 cycles of the first line chemotherapy were included into the study. Prolongation of chemotherapy (8 cycles) was performed to patients who responded to chemotherapy. Characteristics of patients, disease, and treatment are presented in Table 2.

The last date of follow-up was December 31^st ^2003. The dates of death and the cause of death were obtained from the records of the Lithuanian Cancer Registry. No patients were found alive by December 31^st ^2003, and no other causes of death rather than ovarian cancer were certified in the death certificates. Two endpoints have been used in the study – the date of death (overall survival) and the date of disease progression (progression-free survival). Overall and progression-free survival rates and survival medians were calculated using life tables and the Kaplan-Meier method. The univariate and multivariate Cox proportional hazards modelling was applied in order to evaluate the significance of prognostic factors (age, morphology, treatment (adjuvant or neoadjuvant chemotherapy), the optimality of surgery, and the duration of first-line chemotherapy) on the overall survival and disease-free survival. The survival estimates and the Cox proportional hazard modelling were performed using procedures of the STATA (version 7). The hazard ratio was calculated in order to establish the difference in the degree of survival between the groups, and to account for differences that might have been the effects of tumour stage, treatment, age, and other patient characteristics. The ratio of less than one indicated fewer deaths compared to the reference group, whereas the ratio greater than one indicated the opposite. The log-rank test was used to investigate the difference in overall and disease-free survival rates between the studied groups. Mantel-Haenszel estimate was used to estimate the odds ratio of cytoreductive surgery followed by neoadjuvant treatment. Estimates were also controlled by age and stage. All the statistical tests were two-tailed, and *p*-values of less than 0.05 were considered to be significant. The study was approved by the local ethical committee (approval number – BE-2-53-A).

## Results

**Figure 1:**
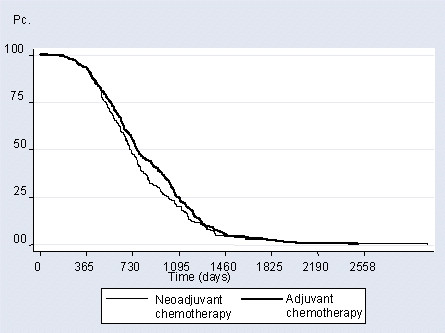
Overall survival for patients with stage III and IV ovarian cancer in the neoadjuvant chemotherapy and adjuvant chemotherapy groups.

**Figure 2:**
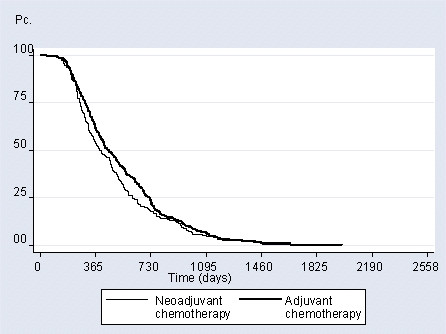
Progression-free survival for patients with stage III and IV ovarian cancer in the neoadjuvant chemotherapy and adjuvant chemotherapy groups.

In the group of patients who underwent neoadjuvant chemotherapy, the median overall survival was 712 days (23.7 months), [95% CI 668–769], and the median progression-free survival – 400 days (13.3 months), [95% CI 347–465]. In the group of patients who received conventional treatment (adjuvant chemotherapy), the median overall survival was 762 days (25.4 months), [95% CI 730–783], and the median progression-free survival – 451 [95% CI 410–501] days (15 months). No statistically significant difference was observed in the median overall survival between the group treated with neoadjuvant chemotherapy and the conventionally treated group (*p *= 0.1310) (Figure 1). No statistical difference was found in the median progression-free survival between neoadjuvant and adjuvant chemotherapy groups (*p *= 0.078) (Figure 2).

The median overall survival in stage III patients treated with neoadjuvant chemotherapy was 778 days (25.9 months) [95% CI 723–850], and in those treated conventionally – 879 days (29.3 months) [95% CI 780–951] (*p *= 0.2508). The median progression-free survival in stage III patients treated with neoadjuvant chemotherapy was 471 days (15.7 months) [95% CI 388–515], and in those treated with adjuvant chemotherapy – 524 days (17.5 months) [95% CI 478–567] (*p *= 0.1299). The median survival in stage IV patients treated with neoadjuvant chemotherapy was 463 days (15.4 months) [95% CI 403–503], and in those treated conventionally – 446 days (14.9 months) [95% CI 411–502] (*p *= 0.6108). The median progression-free survival in stage IV patients treated with neoadjuvant chemotherapy was 261 days (8.7 months) [95% CI 237–291], and in those treated with adjuvant chemotherapy – 245 days (8.2 months) [95% CI 213–287] (*p *= 0.1817).

There were no significant differences in overall survival between both treatment groups in case of serous (*p *= 0.396), endometrioid (*p *= 0.197), and mucinous tumours (*p *= 0.256). No significant differences in progression-free survival between neoadjuvant and standard treatment groups were found in case of serous (*p *= 0.299), endometrioid (*p *= 0.098), and mucinous tumours (*p *= 0.509) either.

No statistical difference was observed in the overall survival (*p *= 0.065) or in the progression-free survival (*p *= 0.094) between the group treated with neoadjuvant chemotherapy and the conventionally treated group for those patients who underwent optimal cytoreduction. No statistical difference was observed in the overall survival (*p *= 0.117) or in the progression-free survival (*p *= 0.791) between the group treated with neoadjuvant chemotherapy and the conventionally treated group for those patients who underwent not optimal cytoreduction. The analysis of subgroups of patients with different histological subtypes of tumours showed no significant difference in overall and progression-free survival for optimally and suboptimally debulked patients between neoadjuvant chemotherapy and standard treatment.

After the neoadjuvant chemotherapy, optimal cytoreductive surgery was performed in 134 patients (63%). Optimal cytoreductive surgery was also performed in 242 conventionally treated patients (67%). Similar rates of optimal cytoreductive surgery were established in the neoadjuvant chemotherapy and conventional therapy groups for stage III patients younger than 65 years of age (79% vs. 78%). The possibility to perform optimal cytoreduction for stage III patients up to 65 years of age did not differ when choosing either adjuvant or neoadjuvant treatment (OR = 1.06 [0.58–2.00], *p *= 0.839). Similar rates of optimal cytoreductive surgery were established in the neoadjuvant chemotherapy and conventional therapy groups for stage III patients older than 65 years of age (66% vs. 62%). Possibilities to perform optimal surgery for stage III patients aged over 65 years in comparison with the younger ones decreased – only two thirds of all patients aged over 65 years were debulked optimally. The possibility of optimal surgery was a bit greater after neoadjuvant chemotherapy, comparing to adjuvant chemotherapy, but no statistically significant difference between these treatment methods was found (OR = 1.18 [0.58–2.42], *p *= 0.626). Similar rates of optimal cytoreductive surgery were established in the neoadjuvant chemotherapy and conventional therapy groups for stage IV patients younger than 65 years of age (36% vs. 54%). The possibility to undergo optimal surgery did not significantly differ for stage IV patients younger than 65 years of age irrespectively of whether the treatment was started with surgery or with adjuvant chemotherapy (OR = 0.48 [0.12–1.83], *p *= 0.226). Similar rates of optimal cytoreductive surgery were established in the neoadjuvant chemotherapy and conventional therapy groups for stage IV patients older than 65 years of age (12% vs. 15%). The possibility of optimal surgery for the patients aged over 65 years remained the same in both groups irrespectively of whether the treatment was started with surgery or with neoadjuvant chemotherapy (OR = 0.77 [0.11–4.52], *p *= 0.741).

The possibility to perform optimal surgery after neoadjuvant chemotherapy did not significantly increase for patients with serous (OR = 0.37 [0.44–1.54], *p *= 0.543), endometrioid (OR = 0.22 [0.39–1.78], *p *= 0.639), and mucinous tumours (OR = 0.24 [0.42–1.68], *p *= 0.622).

In multivariate analysis, the independent prognostic factors for better overall survival of patients with stage III and IV ovarian cancer were optimal cytoreductive surgery (*p *= 0.0001), age (<65 years) (*p *= 0.002) and serous carcinomas (*p *= 0.015). Univariate Cox analysis showed that neoadjuvant chemotherapy did not influence the overall (HR = 0.89, CI [0.74–1.08], *p *= 0.252) and progression-free survival (HR = 0.86, CI [0.71–1.04], *p *= 0.131) for patients with stage III ovarian cancer. According to the estimates of the multivariate Cox analysis, age, stage, tumour morphology, optimal surgery, and the number of the first-line chemotherapy cycles were similar predictors of overall (HR = 0.91, CI [0.75–1.11], p = 0.401) and progression-free survival (HR = 0.90, CI [0.74–1.09], p = 0.299) for the adjuvant and neoadjuvant chemotherapy groups of patients with stage III ovarian cancer.

The results of univariate and multivariate Cox analyses for patients with stage IV ovarian cancer were similar to those in patients with stage III ovarian cancer. Univariate Cox analysis showed that the application of neoadjuvant chemotherapy, comparing to adjuvant chemotherapy, did not influence the overall (HR = 1.10, CI [0.74–1.64], *p *= 0.614) and progression-free survival (HR = 1.30, CI [0.87–1.94], *p *= 0.186). According to the estimates of the multivariate Cox analysis, age, stage, tumour morphology, optimal surgery, and the number of the first-line chemotherapy cycles were similar predictors of overall (HR = 1,15, CI [0.76–1.75], p = 0.494) and progression-free survival (HR = 1.36, CI [0.90–2.08], p = 0.140) for the adjuvant and neoadjuvant chemotherapy groups of patients with stage IV ovarian cancer.

## Discussion

Surgery influences the survival of patients with advanced ovarian cancer [23]. Vergote I. et al. showed that the median survival of patients is increased only after a complete removal of a tumour (tumour mass after cytoreductive surgery is <1 g) [24]. Optimal cytoreductive surgery is possible in only to 40–50% of patients with advanced stage ovarian cancer [3]. Five-year survival after not optimal cytoreductive surgery does not exceed 10% [25]. Instead of standard treatment by cytoreductive surgery and adjuvant chemotherapy, patients with advanced ovarian cancer are also treated with neoadjuvant chemotherapy (drug-induced preoperative cytoreduction), which in the opinion of some investigators can create better conditions for optimal cytoreductive surgery. It is believed that such treatment with neoadjuvant chemotherapy improves the survival of patients. Neoadjuvant chemotherapy makes more conservative surgery feasible and, for patients with poor performance status, decreases the risk of postoperative complications. Unfortunately, up to now no randomized studies evaluating the efficacy of neoadjuvant chemotherapy in patients with advanced ovarian cancer have been conducted. Some published studies are mainly retrospective and numbers of patients treated with neoadjuvant chemotherapy and analysed in these studies are small.

The survival of patients treated with neoadjuvant chemotherapy following cytoreductive surgery is similar to those in whom treatment was started with cytoreductive surgery [13,14,19,20,26,27]. Some studies showed that neoadjuvant chemotherapy increases the overall or progression-free survival [8-11]. In contrast to those data, the results of our study showed that the rate of optimal surgery and survival of patients with ovarian cancer did not increase after neoadjuvant chemotherapy. Nevertheless, more advanced tumours in the group of patients who underwent neoadjuvant chemotherapy, and similar overall and disease-free survival results in both treatment groups allow for the assumption that neoadjuvant chemotherapy might have a positive impact in patients with advanced ovarian carcinomas. Prospective randomized clinical trials are needed to clarify the validity of neoadjuvant chemotherapy.

Usually, neoadjuvant chemotherapy is administered expecting that inoperable tumours will decrease in size, and it will be possible to perform optimal surgery leading to better survival results. Only a few studies showed significantly better tumour resection rates after neoadjuvant chemotherapy comparing to conventional treatment [8,17]. We established that independently of patients' age, the possibility to perform optimal surgery after neoadjuvant chemotherapy did not increase in patients with stage III and IV ovarian cancer. A similar number of optimal cytoreductive procedures in both groups shows that neoadjuvant chemotherapy administered in the presence of more extensive ovarian cancer might increase the possibility to perform optimal surgery. It was assumed that neoadjuvant chemotherapy could improve the chance of optimal surgery and survival for selected patients who cannot be debulked optimally using primary cytoreductive surgery [11]. Currently, there are no established criteria that would help to determine which treatment – neoadjuvant or adjuvant chemotherapy – should be administered to patients with advanced ovarian cancer. Future investigations are needed in order to identify patients who will benefit from neoadjuvant chemotherapy.

## Conclusion

There was no difference in progression-free or overall survival, although patients receiving neoadjuvant chemotherapy had a more extensive disease. The rate of optimal cytoreductive surgery was not statistically different between the neoadjuvant and adjuvant chemotherapy groups; however, neoadjuvant chemotherapy was administered in cases of more extensive ovarian cancer. Multivariate analysis did not prove that neoadjuvant chemotherapy could be an independent prognostic factor for survival, and the findings need to be investigated in future prospective randomised studies.

## Competing interests

The author(s) declare that they have no competing interests.

## Authors' contributions

A.I. initiated the study, participated in its design and coordination, carried out the study, and drafted the manuscript. A.S. carried out the study. E.J. participated in the design and coordination of the study. J.K. performed the statistical analysis. R.N. participated in the coordination of the study. E.S. participated in the design of the study and helped to draft the manuscript. S.K. participated in the coordination of the study. All authors read and approved the final manuscript.

## Pre-publication history

The pre-publication history for this paper can be accessed here:



## Figures and Tables

**Table 1:** Retrospective studies in which patients with advanced ovarian cancer were treated with neoadjuvant chemotherapy followed by cytoreductive surgery.

**Authors**	**No. of patients treated with neoadjuvant chemotherapy**	**Main conclusion**
Kuhn W et al. (8)	37	Better median survival in the group treated with neoadjuvant chemotherapy compared with the group treated with primary cytoreductive surgery
Onnis A et al. (9)	88	In 46% of operated patients, the residual tumour was < 2 cm. An improvement in the quality of surgery and disease-free period was observed, while survival rate did not improve when comparing the neoadjuvant chemotherapy group to the group that received adjuvant chemotherapy
Shimizu Y et al. (10)	74	In 46% of operated patients, the residual tumour was < 2 cm. Survival for the optimally debulked neoadjuvant chemotherapy group was significantly higher than that for the optimally debulked adjuvant chemotherapy group
Vergote I et al. (11)	75	Crude survival was higher when selecting about one-half of patients for neoadjuvant chemotherapy
Surwit E. et al. (12)	29	Median survival was 22 months
Schwartz PE et al. (13)	59	No statistical difference was observed in overall and progression-free survival between the group treated with neoadjuvant chemotherapy and the conventionally treated group
Jacob JH et al. (14)	22	The same survival as in 18 matched controls
Lim JT et al. (15)	30	Neoadjuvant chemotherapy can make patients operable
Lawton FG et al. (16)	36	78% of patients were treated with neoadjuvant chemotherapy, of whom in 89% the residual tumour was < 2 cm
Ansquer Y et al. (17)	54	Better survival of patients treated with neoadjuvant chemotherapy compared to patients with non-debulked tumours
Recchia F et al. (18)	34	Median survival was 28 months for stage IV patients
Loizzi V et al. (19)	30	Survival in the neoadjuvant and adjuvant chemotherapy groups was similar
Kayikcioglu F et al. (20)	45	Primary neoadjuvant chemotherapy followed by cytoreductive surgery did not appear to worsen the prognosis
Morice P et al. (21)	57	Survival rates were similar in patients who underwent interval debulking surgery or primary debulking surgery
Ivanov S et al. (22)	46	The five-year survival rate following the neoadjuvant chemotherapy was the same as in case of conventional chemotherapy

**Table 2:** Characteristics of patients, disease, and treatment

**Feature**	**Neoadjuvant chemotherapy**	**Adjuvant chemotherapy**	**Total**
**Number of patients**	213	361	574

**Stage:**			
III	165	305	470
IV	48	56	104

**Age by years:**			
<65	123	236	359
>65	90	125	215

**Morphology:**			
Serous	84	135	219
Mucinous	48	67	115
Endometrioid	49	118	167
Other types (clear cell, undifferentiated and mixed)	32	41	73

**Cytoreductive surgery:**			
Optimal	134	242	376
Suboptimal	79	119	198

**Number of chemotherapy cycles**			
6 cycles	109	244	353
8 cycles	104	117	221
